# A Machine-Learning Approach for Dynamic Prediction of Sepsis-Induced Coagulopathy in Critically Ill Patients With Sepsis

**DOI:** 10.3389/fmed.2020.637434

**Published:** 2021-01-21

**Authors:** Qin-Yu Zhao, Le-Ping Liu, Jing-Chao Luo, Yan-Wei Luo, Huan Wang, Yi-Jie Zhang, Rong Gui, Guo-Wei Tu, Zhe Luo

**Affiliations:** ^1^Department of Blood Transfusion, The Third Xiangya Hospital of Central South University, Changsha, China; ^2^College of Engineering and Computer Science, Australian National University, Canberra, ACT, Australia; ^3^Department of Critical Care Medicine, Zhongshan Hospital, Fudan University, Shanghai, China; ^4^Department of Critical Care Medicine, Xiamen Branch, Zhongshan Hospital, Fudan University, Xiamen, China

**Keywords:** sepsis-induced coagulopathy, dynamic prediction, machine learning, Logistic Regression, external validation, model interpretation

## Abstract

**Background:** Sepsis-induced coagulopathy (SIC) denotes an increased mortality rate and poorer prognosis in septic patients.

**Objectives:** Our study aimed to develop and validate machine-learning models to dynamically predict the risk of SIC in critically ill patients with sepsis.

**Methods:** Machine-learning models were developed and validated based on two public databases named Medical Information Mart for Intensive Care (MIMIC)-IV and the eICU Collaborative Research Database (eICU-CRD). Dynamic prediction of SIC involved an evaluation of the risk of SIC each day after the diagnosis of sepsis using 15 predictive models. The best model was selected based on its accuracy and area under the receiver operating characteristic curve (AUC), followed by fine-grained hyperparameter adjustment using the Bayesian Optimization Algorithm. A compact model was developed, based on 15 features selected according to their importance and clinical availability. These two models were compared with Logistic Regression and SIC scores in terms of SIC prediction.

**Results:** Of 11,362 patients in MIMIC-IV included in the final cohort, a total of 6,744 (59%) patients developed SIC during sepsis. The model named Categorical Boosting (CatBoost) had the greatest AUC in our study (0.869; 95% CI: 0.850–0.886). Coagulation profile and renal function indicators were the most important features for predicting SIC. A compact model was developed with an AUC of 0.854 (95% CI: 0.832–0.872), while the AUCs of Logistic Regression and SIC scores were 0.746 (95% CI: 0.735–0.755) and 0.709 (95% CI: 0.687–0.733), respectively. A cohort of 35,252 septic patients in eICU-CRD was analyzed. The AUCs of the full and the compact models in the external validation were 0.842 (95% CI: 0.837–0.846) and 0.803 (95% CI: 0.798–0.809), respectively, which were still larger than those of Logistic Regression (0.660; 95% CI: 0.653–0.667) and SIC scores (0.752; 95% CI: 0.747–0.757). Prediction results were illustrated by SHapley Additive exPlanations (SHAP) values, which made our models clinically interpretable.

**Conclusions:** We developed two models which were able to dynamically predict the risk of SIC in septic patients better than conventional Logistic Regression and SIC scores.

## Introduction

Sepsis, defined as life-threatening organ dysfunction caused by a dysregulated host response to infection, remains the first leading cause of mortality in critically ill patients (1, 2). Coagulopathy is one of the major complications of sepsis, leading to a higher risk of thrombosis, the deterioration of organ failure, and an increased mortality rate (3–6). However, the usefulness of anticoagulant therapies has not been confirmed in septic patients (7, 8). Recent observational studies and subgroup analyses of large-scale randomized controlled trials revealed that anticoagulant therapies might result in a significant reduction in mortality risk and improved outcome in septic patients with coagulopathy (9–12). In contrast, anticoagulant therapies in patients without coagulopathy should be avoided due to the increased risk of bleeding with no survival benefit (11, 13). Furthermore, some drugs commonly administered in septic patients, such as linezolid and vancomycin, may alter coagulation function through various mechanisms and should be used with caution in patients with a high risk of coagulopathy (14). These study results have heightened the need for early identification of coagulopathy in septic patients in a timely way.

Sepsis-induced coagulopathy (SIC) criteria were developed by members of the Scientific and Standardization Committee (SSC) on Disseminated Intravascular Coagulation (DIC) of the International Society of Thrombosis and Haemostasis (ISTH) in 2017 (15) (Supplementary Table 1). The criteria are a scoring system designed to identify patients with “sepsis and coagulation disorders.” SIC is defined as a score ≥ 4. It was found that the mortality rate increased as the SIC score rose and exceeded 30% at a score of 4 (15). Compared with DIC, SIC is more relevant for the updated Sepsis-3 criteria (1, 16). In addition, observational evidence has shown that SIC preceded DIC in most cases (17, 18). As a result, the new guideline in 2019 recommended that septic patients with thrombocytopenia (platelet count <150 × 10^9^/L) should be screened, first using SIC diagnostic criteria and then using ISTH DIC diagnostic criteria (16). However, the SIC score mainly serves as a diagnostic system; there is still a lack of reliable predictive tools for SIC in clinical practice.

In recent years, the emergence of new machine-learning algorithms has enabled us to predict disease events dynamically based on huge and complicated clinical information. Advanced machine-learning models can fit high-order relationships between covariates and outcomes, and therefore, they excel in the analysis of complex signals in data-rich environments (19–22). The aims of this study were to develop and validate to develop and validate machine-learning models for the early dynamic prediction of SIC, and to assess the risk features by interpreting the final model.

## Materials and Methods

### Source of Data

We conducted this retrospective study based on two sizeable critical care databases the Medical Information Mart for Intensive Care (MIMIC)-IV (23) and the eICU Collaborative Research Database (eICU-CRD) (24). The MIMIC-IV database is an updated version of MIMIC-III and currently contains comprehensive and high-quality data of patients admitted to intensive care units (ICUs) at the Beth Israel Deaconess Medical Center between 2008 and 2019. The other database, eICU-CRD, is a multicenter database comprising de-identified health data associated with over 200,000 admissions to ICUs across the United States between 2014 and 2015. One author (QZ) obtained access to both databases and was responsible for data extraction. The study was reported according to the recommendations of the Transparent Reporting of a multivariable prediction model for Individual Prognosis Or Diagnosis (TRIPOD) statement (25).

### Selection of Participants

In MIMIC-IV, patients who fulfilled the definition of sepsis between 2008 and 2019 were included. According to the Sepsis-3 criteria, sepsis was defined as a suspected infection combined with an acute increase in Sequential Organ Failure Assessment (SOFA) score ≥ 2 (1). Patients with prescriptions of antibiotics and sampling of bodily fluids for microbiological culture were considered to have suspected infection. In line with previous research, when the antibiotic was given first, the microbiological sample must have been collected within 24 h; when the microbiological sampling occurred first, the antibiotic must have been administered within 72 h (26). Hourly SOFA was evaluated based on the clinical and laboratory data. In eICU-CRD, microbiology data were not well populated due to the limited availability of microbiology interfaces; instead, infection was identified according to documented diagnosis.

Only patients who were older than 18 years and stayed in the ICU for more than 24 h were included. No patients were excluded due to missing values. We made no attempt to estimate the sample size of the study; instead, all eligible patients in MIMIC-IV and eICU-CRD were included to maximize the statistical power of the predictive model.

### Outcome (SIC)

We annotated patients' every day when the sepsis definition was fulfilled with their current coagulation state according to the SIC criteria, as recommended (16). Specifically, the worst daily values of SIC-related indicators were extracted. Then daily repeated scoring was performed. A patient was annotated as SIC positive if he or she had a SIC score ≥ 4 on that day.

### Predictors of SIC

Clinical and laboratory variables were extracted during sepsis. For some variables with multiple measurements, average values were assessed. For the prediction of SIC, 88 variables were collected (Supplementary Table 2), including patient characteristics (age, gender, ethnicity, admission type), vital signs (respiratory rate, blood pressure, heart rate, SpO_2_, and temperature), laboratory data (blood gas, routine blood analysis, liver function, renal function, and coagulation profile), transfusion (red blood cells, platelets, and fresh frozen plasma) and urine output. Comorbidities were also collected based on the recorded International Classification of Diseases (ICD)-9 and ICD-10 codes, including hypertension, diabetes mellitus, chronic obstructive pulmonary disease, congestive heart failure, myocardial infarction, chronic kidney disease, leukemia, stroke, cancer, and liver disease. Lastly, medications such as heparin, antibiotics and vasopressors, continuous renal replacement therapy (CRRT), and mechanical ventilation (MV) were collected.

### Statistical Analysis

Baseline characteristics on the first sepsis day were compared between SIC and non-SIC groups in MIMIC-IV. Values are presented as the means [standard deviations] (if normal) or medians [interquartile ranges] (if non-normal) for continuous variables, and total numbers [percentages] for categorical variables. Comparisons were made using the Student *t*-test or rank-sum test for continuous variables, and the Chi-square test or Fisher's exact test for categorical variables, as appropriate.

**Figure 1:**
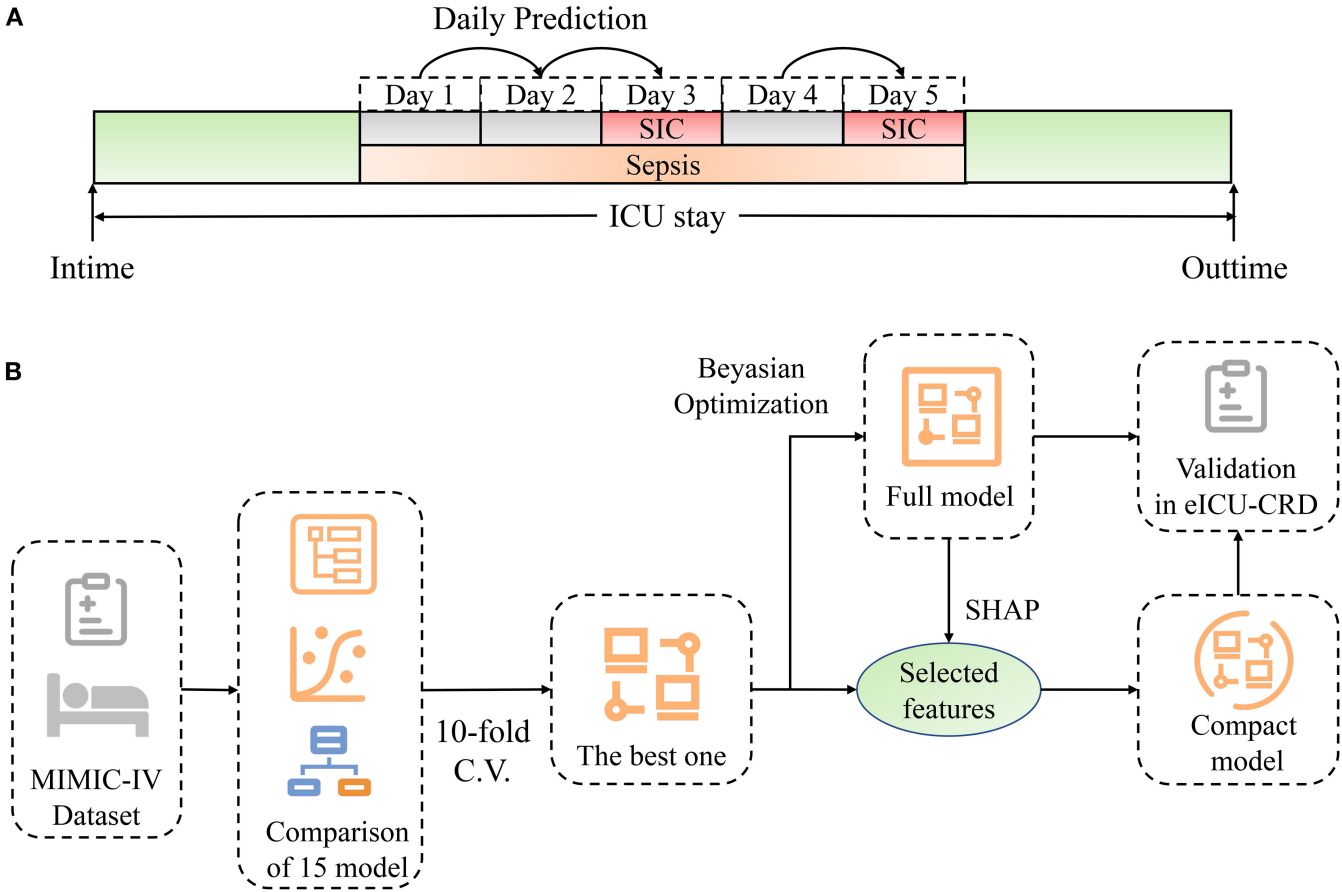
Schematic illustration of the study design. **(A)** Design of dynamic prediction in our study. Daily assessment was performed from the time when sepsis was diagnosed. If SIC criteria were not fulfilled, the risk of SIC the next day was predicted by our model. Prediction stopped when SIC was diagnosed, and restarted when patients recovered from SIC. **(B)** Schematic illustration of model development. We compared the discrimination of 15 machine-learning models using 10-fold cross-validation. The model with the best accuracy and greatest AUC was chosen. Fine-grained hyperparameter adjustment was performed using Bayesian Optimization. Fifteen features were selected according to their SHAP values and clinical availability. A compact model was developed based on the selected features. Lastly, these two models were validated in eICU-CRD. ICU, intensive care unit; SIC, sepsis-induced coagulopathy; SHAP, SHapley Additive exPlanations; MIMIC-IV, Medical Information Mart for Intensive Care-IV; C.V., cross-validation; eICU-CRD, the eICU Collaborative Research Database.

As shown in Figure 1A, our model generated a continuous prediction score based on the above-mentioned 88 variables on each day when patients were diagnosed with sepsis. The scores assessed the risk of SIC in the following day. Prediction was not performed if SIC criteria were already fulfilled on that day; when the patients recovered from SIC, our model then restarted to predict if they still had sepsis. None of the imputation methods were used for advanced boosting machine-learning methods as they automatically handle missing values; in contrast, missing values were imputed using the median values for continuous variables or mode values for categorical values when training other models. As shown in Figure 1B, we preliminarily compared the prediction performance of 15 algorithms using the *PyCaret* Python package (version 1.0.0), an open-sourced, automated machine-learning workflow. The assessment process was performed using 10-fold cross-validation. Accuracy and area under the receiver operating characteristic curve (AUC) were calculated on each fold and pooled to evaluate each model. The algorithm with the highest accuracy and the largest AUC was selected. Then, we performed fine-grained hyperparameter adjustment for the potential model using the Bayesian Optimization Algorithm. This algorithm is an efficient constrained global optimization tool, which was performed using the functions of the *bayes_opt* Python package (version 1.2.0) (27). The optimized model was the best model for SIC prediction in this study and was defined as the full model.

The effects of features on prediction scores were measured using the functions of the *SHapley Additive exPlanations (SHAP)* Python package (version 0.32.1), which assessed the importance of each feature using a game-theoretic approach based on the validation set (28). We selected 15 features which had great importance and were as easy as possible to collect in the clinical setting (Supplementary Table 2). A compact model was then trained for SIC prediction based on the selected features. Although this model was not as accurate as the full model, it might be more practical in clinical settings.

External validation of the full and compact models was performed in eICU-CRD. The median and 95% confidence intervals of AUC were calculated using the Bootstrap Resampling technique with 1,000 iterations. Conventional Logistic Regression and the SIC scoring system were assessed to predict the risk of SIC and were compared with our models in both internal and external validations.

All analyses were performed using Python (version 3.7.6), and *p* < 0.01 was considered statistically significant.

## Results

### Baseline Characteristics

**Figure 2:**
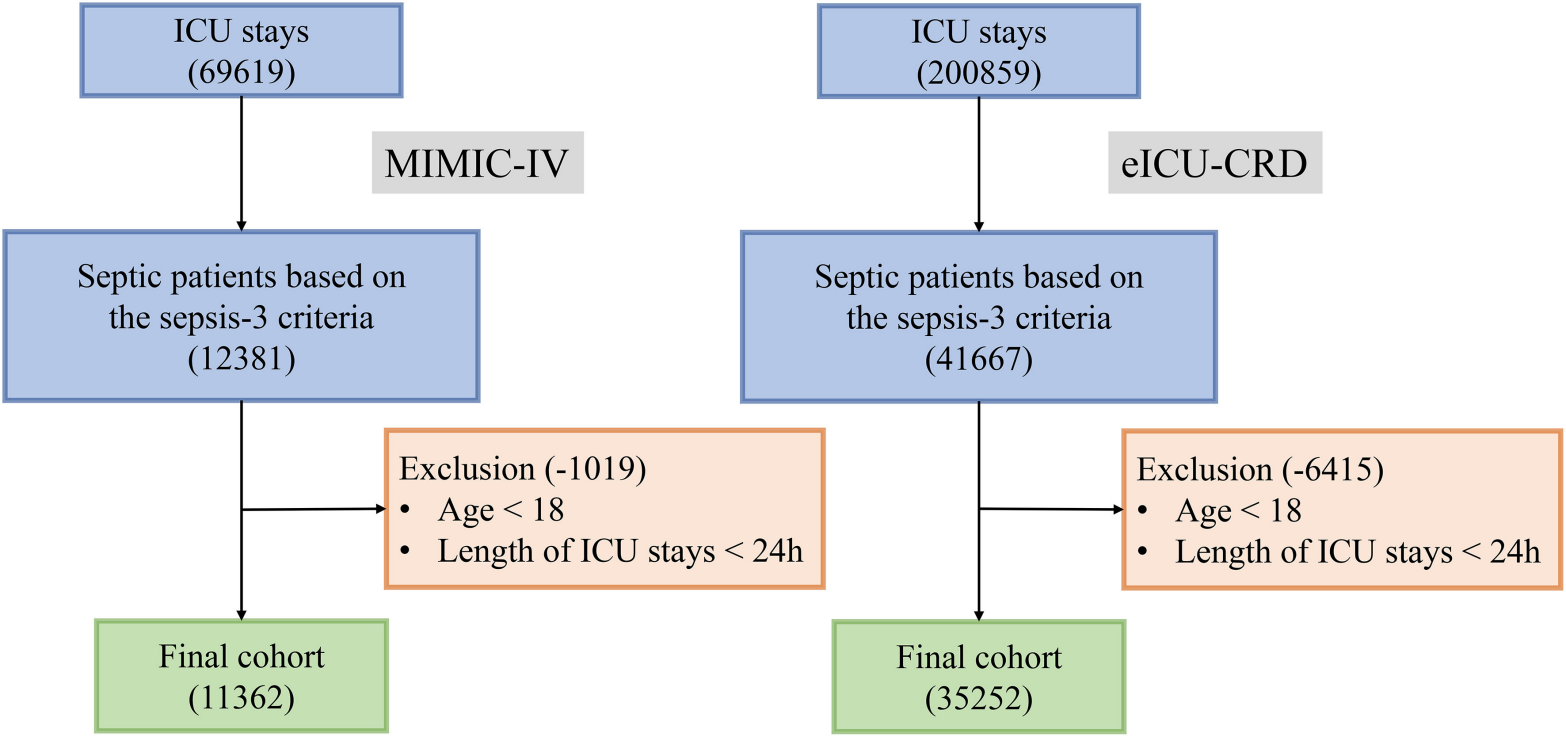
Flow chart of patient selection.

As shown in Figure 2, of 12,381 septic patients in MIMIC-IV, 11,362 were included in the final cohort. A total of 6,744 patients developed SIC during sepsis, and 4,618 patients did not. A cohort of 35,252 septic patients in eICU-CRD was included as external dataset.

Variable values on the first day of sepsis in MIMIC-IV were analyzed; the differences in characteristics were compared (Supplementary Table 3). The SIC group had a higher rate of comorbidities, higher SAPS-II scores (44 [35, 54] vs. 37 [30, 45]; *p* < 0.001), higher SOFA scores (6 [4, 9] vs. 4 [3, 5]; *p* < 0.001), longer prothrombin time (PT) (16.9 [14.3, 21.8] vs. 13.0 [11.9, 14.1]; *p* < 0.001), less urine output (790 [300, 1,545] vs. 1,205 [605, 2,015]; *p* < 0.001), higher rates of linezolid (2.9 vs. 1.7%; *p* < 0.001), vancomycin (55.6 vs. 46.0%; *p* < 0.001), CRRT (5.0 vs. 0.6%; *p* < 0.001), vasopressors (46.8 vs. 23.2%; *p* < 0.001) and MV (50.3 vs. 40.6%; *p* < 0.001), and higher 28-day mortality (27.0 vs. 10.8%; *p* < 0.001) than the non-SIC group. The length of hospital stay was also longer in the SIC group than in the non-SIC group (14.4 [7.9, 26.7] vs. 10.9 [6.5, 19.5], *p* < 0.001).

### Comparison of 15 Models

Daily data were extracted, and 16,183 samples for prediction in MIMIC-IV were created. Of these samples, 1,489 were labeled as positive (SIC the next day), 14,694 were labeled as negative (still non-SIC the next day). The prediction performances of the various models are listed in Table 1. As shown, Logistic Regression had an acceptable performance (accuracy: 0.908; AUC: 0.746). Ensemble learning algorithms had better accuracy and larger AUC than others, such as Categorical Boosting (CatBoost) (accuracy: 0.913; AUC: 0.841), Light Gradient Boosting (accuracy: 0.912; AUC: 0.835) and Random Forest Classifier (accuracy: 0.909; AUC: 0.760). The CatBoost model had the most powerful discrimination for predicting SIC risk, and we optimized this model in the next step.

**Table 1:** Performance of different models in internal validation.

	**Model**	**Accuracy**	**AUC**
1	CatBoost Classifier	0.913 (±0.004)	0.841 (±0.025)
2	Light Gradient Boosting	0.912 (±0.005)	0.835 (±0.024)
3	Extreme Gradient Boosting	0.912 (±0.004)	0.837 (±0.025)
4	Gradient Boosting Classifier	0.911 (±0.005)	0.832 (±0.023)
5	Extra Trees Classifier	0.911 (±0.002)	0.819 (±0.032)
6	Random Forest Classifier	0.909 (±0.002)	0.760 (±0.022)
7	Ridge Classifier	0.908 (±0.003)	0.753 (±0.031)
8	Logistic Regression	0.908 (±0.002)	0.746 (±0.030)
9	K Neighbors Classifier	0.904 (±0.001)	0.611 (±0.040)
10	Ada Boost Classifier	0.902 (±0.003)	0.804 (±0.029)
11	Linear Discriminant Analysis	0.902 (±0.003)	0.796 (±0.027)
12	Multi-Level Perceptron	0.883 (±0.004)	0.754 (±0.022)
13	Decision Tree Classifier	0.861 (±0.003)	0.593 (±0.019)
14	SVM – RBF Kernel	0.859 (±0.004)	0.777 (±0.015)
15	Naive Bayes	0.805 (±0.005)	0.756 (±0.031)

*Models are ordered according to their accuracy*.

*AUC, area under receiver operating characteristic curve; CatBoost, Categorical Boosting; SVM, support vector machine; RBF, Radial Basis Function*.

### Full and Compact Models

**Figure 3:**
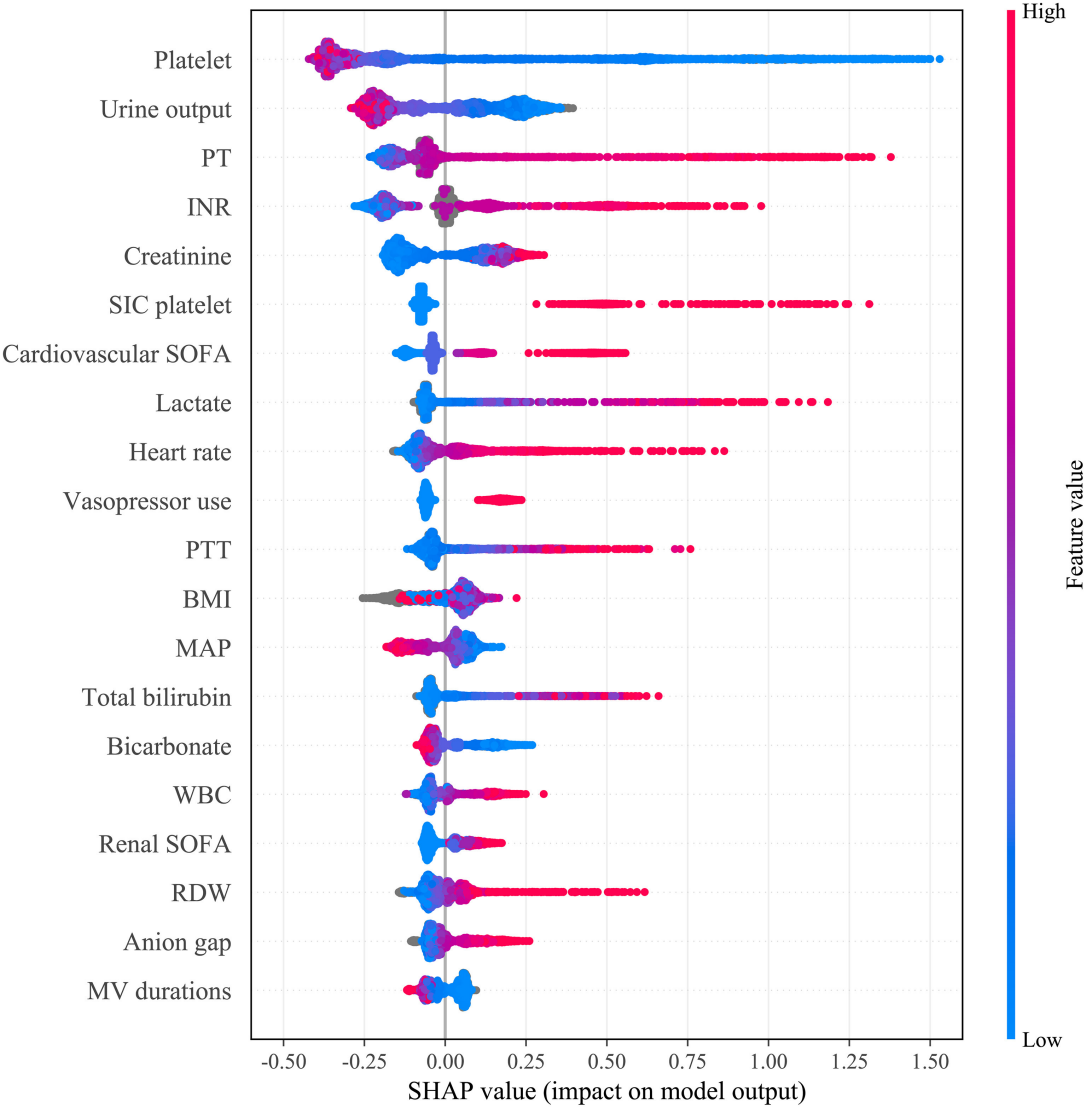
Distribution of the impact each feature had on the full model output estimated using the SHapley Additive exPlanations (SHAP) values. The plot sorts features by the sum of SHAP value magnitudes over all samples. The color represents the feature value (red high, blue low). The x axis measures the impact on the model output (right positive, left negative). Taking the feature platelet as an example, red points are on the left whereas blue points are on the right. This means prediction scores will be smaller when patients have a low level of platelets. PT, prothrombin time; INR, international normalized ratio; SIC, sepsis-induced coagulopathy; SIC platelet, platelet term in the SIC score; SOFA, sequential organ failure assessment; PTT, Partial Thromboplastin Time; BMI, body mass index; MAP, mean arterial pressure; WBC, white blood cell count; RDW, red cell distribution width; MV, mechanical ventilation.

**Figure 4:**
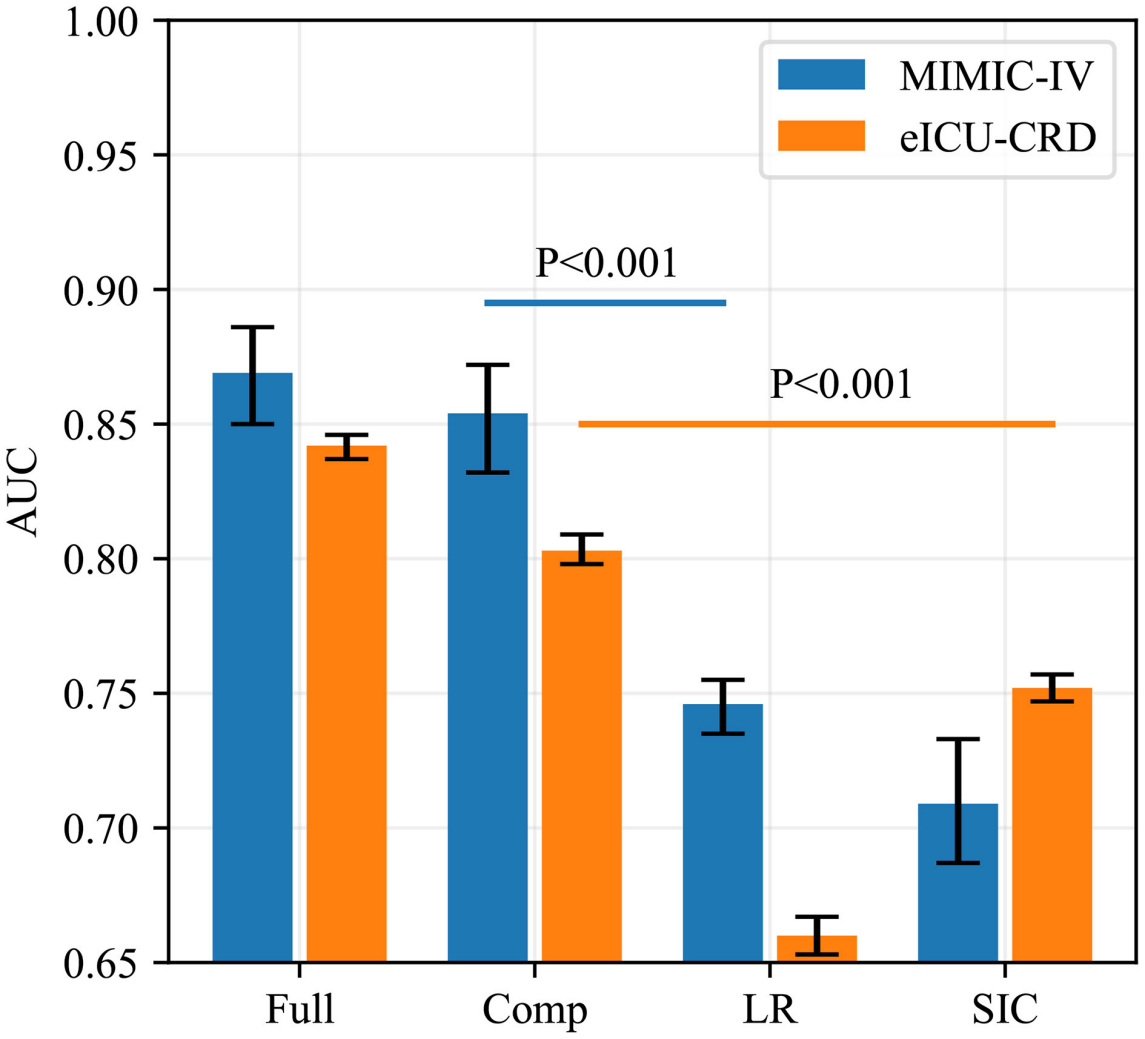
AUCs of four predictive methods in internal (MIMIC-IV) and external (eICU-CRD) validations. AUCs of our two models, Logistic Regression and SIC scores were assessed using the Bootstrap Resampling technique with 1,000 iterations. The heights of the bars represent the median AUCs, while the error bars represent the 95% confidence intervals. Full, the full model; Comp, the compact model; LR, Logistic Regression; SIC, the sepsis-induced coagulopathy criteria; AUC, area under receiver operating characteristic curve; MIMIC-IV, Medical Information Mart for Intensive Care-IV; eICU-CRD, the eICU Collaborative Research Database.

Fifteen iterations of Bayesian optimization were performed. The hyperparameter search domains and final settings are listed in Supplementary Table 4. The optimized CatBoost model had the greatest AUC in our study (0.869; 95% CI: 0.850–0.886). SHAP values were calculated and are plotted in Figure 3. The summary plot sorts features by the sum of SHAP value magnitudes over all samples and shows the distribution of the impact that each feature has on the full model output. As shown, the coagulation profile (platelet, International Normalized Ratio, PT) and renal function indicators (urine output, creatinine) are the most important features for distinguishing the SIC and non-SIC groups. Fifteen features were selected based on their SHAP values and clinical availability. The compact CatBoost model was built based on the selected features. It had a slightly smaller AUC (0.854; 95% CI: 0.832–0.872), but is considered more practical in clinical practice. The medians and 95% confidence intervals of AUCs are plotted in Figure 4 to compare the discrimination of different methods in MIMIC-IV. As shown, our two models outperformed conventional Logistic Regression (0.746; 95% CI: 0.735–0.755) and the SIC scoring system (0.709; 95% CI: 0.687–0.733) in terms of SIC prediction.

### Prediction Performance in eICU-CRD

The results of external validation are shown in Figure 4 ([0.842; 95% CI: 0.837–0.846] for the full model, and [0.803; 95% CI: 0.798–0.809] for the compact model). It can be seen that the SIC scoring system had better predictive power (0.752; 95% CI: 0.747–0.757) than in MIMIC-IV but its AUC was still worse than those of our two models (*p* < 0.001), while Logistic Regression had the poorest generalization ability (0.660; 95% CI: 0.653–0.667). The sensitivity and specificity analysis of the four predictive methods is summarized in Table 2.

**Table 2:** Performance of the final models and SIC scores in internal and external validations.

	**Internal validation (MIMIC-IV)**				**External validation (eICU-CRD)**			
**Model**	**AUC**	**Youden**	**Sensitivity**	**Specificity**	**AUC**	**Youden**	**Sensitivity**	**Specificity**
The full model	0.869	0.577	0.820	0.757	0.842	0.54	0.8	0.741
The compact model	0.854	0.564	0.848	0.716	0.803	0.477	0.745	0.732
Logistic Regression	0.746	0.433	0.753	0.680	0.660	0.230	0.582	0.648
SIC scores	0.709	0.368	0.707	0.661	0.752	0.448	0.655	0.793

*The discrimination of three models (the full model, the compact model and Logistic Regression) and SIC scores were compared in internal and external validations. The full and the compact models were developed in MIMIC-IV, based on all or selected features, respectively. Logistic Regression was developed based on all features. In addition, the current SIC score was used to predict patient's SIC risk the next day. Youden Index, defined as Sensitivity + Specificity − 1, and AUC assessed the performance of different models. All statistics were the median values in 1,000 iterations of the Bootstrap Resampling technique*.

*SIC, Sepsis-induced coagulopathy; AUC, area under receiver operating characteristic curve; MIMIC, Medical Information Mart for Intensive Care; eICU-CRD, the eICU Collaborative Research Database*.

**Figure 5:**
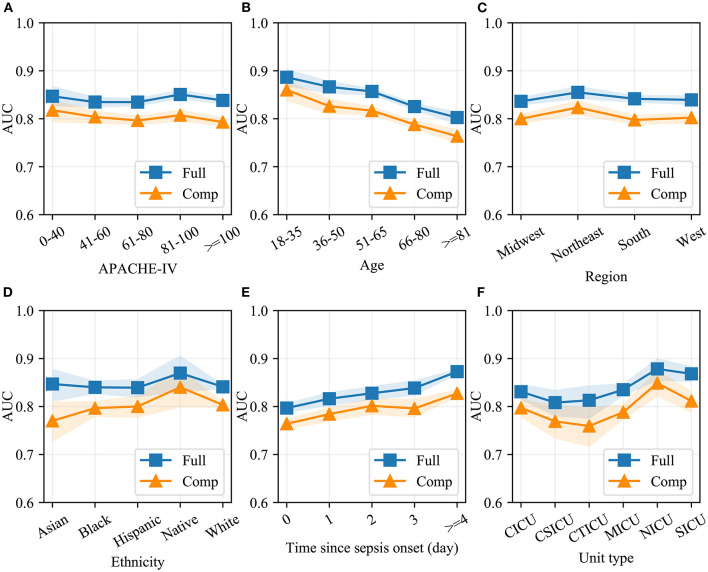
Model performance in different patient cohorts in eICU-CRD. Different validation sets were derived based on APACHE-IV **(A)**, age **(B)**, region of the United States **(C)**, ethnicity **(D)**, time since sepsis onset **(E)** and unit type **(F)**. AUC of the full and the compact models in each set was measured using the Bootstrap Resampling technique. The colored area represents 95% confidence intervals. Full, the full model; Comp, the compact model; AUC, area under receiver operating characteristic curve; APACHE-IV, Acute Physiology and Chronic Health Evaluation-IV; CICU, cardiac intensive care unit; CSICU, cardiac surgical intensive care unit; CTICU, cardiothoracic intensive care unit; MICU, medical intensive care unit; NICU, neuro intensive care unit; SICU, surgical intensive care unit.

Model performance in different patient cohorts in eICU-CRD is shown in Figure 5. As shown, the two models had the greatest AUC for patients who had APACHE-IV scores between 81 and 100, who were younger than 65 years, or who were admitted to the NICU and SICU. The two models maintained good performance over four regions of the United States. In addition, the two models had better discrimination when sepsis lasted for several days. A similar sub-cohort analysis was also performed in MIMIC-IV (Supplementary Figure 1).

### Model Interpretation

**Figure 6:**
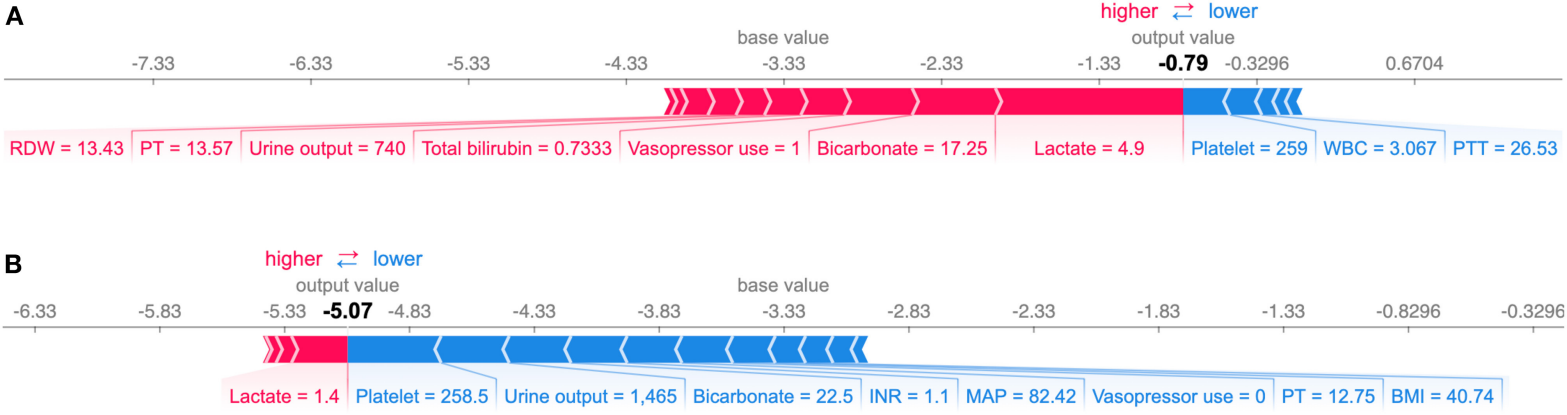
Explanation of the prediction results for specific instances. The base value (−3.33) is the average value of the predictive model; the output values are the predicted SIC risks. The bars in red and blue represent risk factors and protective factors, respectively; longer bars mean greater feature importance. Here, these values are the model outputs before the SoftMax layer, and therefore, they are not equal to the final predicted probabilities. This figure shows the explanation for a high-risk instance **(A)** and a low-risk instance **(B)**. RDW, red cell distribution width; PT, prothrombin time; WBC, white blood cell count; PTT, Partial Thromboplastin Time; INR, international normalized ratio; MAP, mean arterial pressure; BMI, body mass index.

The summary plot of SHAP in Figure 3 provides an overview of the impact of features on the final models. Additionally, the prediction results of two specific instances are explained in Figure 6. The bars in red and blue represent risk factors and protective factors, respectively; longer bars represent greater feature importance. For the example in Figure 6A, although the patient's coagulation profile was normal, she had a poor circulatory status with a high serum lactate level and the vasopressor administration. The model successfully predicted that she would have SIC the next day. For the example in Figure 6B, the patient's condition was more moderate, and our model predicted a low-risk value.

### Website-Based Tool

A website-based tool was established for clinicians to use the compact model, http://www.aimedicallab.com/tool/aiml-sicrisk.html. The SIC risk in the following day can be assessed by using this tool, and interpretation of the prediction result in the instance level will be shown to the user.

## Discussion

To the best of our knowledge, this is first attempt to apply machine-learning models for the dynamic prediction of SIC. Our study developed and validated two variants of dynamic machine-learning models, providing an accurate predictive tool for SIC in sepsis patients.

In this study, we reconfirmed that coagulopathy worsens the clinical outcomes of septic patients (15). As shown in Supplementary Table 3, SIC can lead to a higher mortality rate and longer length of hospital/ICU stay. In addition, SIC patients received more advanced antibiotics (linezolid and vancomycin), implying a more severe state of infection. On the other hand, the administration of these drugs may also alter coagulation function through various mechanisms (29, 30). As a result, early identification of septic patients with high coagulopathy risks is of great importance.

Currently, there is a lack of reliable tools for the early prediction of coagulopathy in septic patients. Our study demonstrated that the family of gradient boosting algorithms, such as CatBoost, Light Gradient Boosting and Extreme Gradient Boosting, can predict SIC with higher accuracy than others. In short, gradient boosting is a powerful machine-learning technique that iteratively trains a weak classifier (e.g., decision tree) to fit residuals of previous models (31). CatBoost, one of gradient boosting algorithms, showed the greatest AUC in our study, partly because it had two main advantages. First, it successfully handles categorical features and deals with them during training instead of preprocessing time (32). This means that categorical features no longer need to be encoded, and a CatBoost model can be developed based on raw data. Another advantage of this algorithm is that it uses a new schema to calculate leaf values when selecting the tree structure. The schema helps to reduce overfitting, a major problem that constrains the generalization ability of machine-learning models (32).

In this study, we developed two variants of CatBoost models that can identify patients with a high risk of SIC and provide clinical decision-makers with more information. As shown in Figure 5, our models had comparable AUCs in different patient cohorts, demonstrating that machine-learning models based on big data have good generalization capability.

In general, based on more valuable variables, models have better discrimination but worse clinical usability. Therefore, in our study, two model variants were developed for different application scenarios. The full model predicted SIC based on 88 clinical variables and achieved the highest AUC in this study. In the external validation, the full model maintained good discrimination with only a slight reduction in AUC. However, it is difficult to collect 88 variables and apply this model. As a result, the full model is recommended in hospitals with a well-designed clinical data system. By contrast, the compact model was trained based on 15 selected variables. Under the condition of ensuring accuracy, it achieved practicality as far as possible. In addition, a website tool was developed to help clinicians use the compact model in clinical practice. By logging on to the website and entering the values of 15 variables, our compact model will give the prediction result, and interpretation of the prediction result will be shown to the user.

By interpreting the full model, it was found that many clinical variables can help to indicate the risk of SIC. In this study, coagulopathy profile was found to be the most important variable in predicting SIC followed by renal function indicators (urine output and creatinine). As shown in Figure 3, patients with poorer renal function (less urine output and higher serum creatinine) tended to have a higher risk of SIC. Also, body mass index (BMI), vital signs (heart rate and mean arterial pressure), laboratory tests (such as lactate and white blood cell count), the use of MV and vasopressors, and SAPS-II scores can help assess the risk of SIC. In addition, prediction results can be illustrated at the instance level, as shown in Figure 6, which makes our model clinically interpretable.

Several limitations of this study should be considered. Firstly, only septic adults in ICUs were included, whereas hospitalized sepsis cases were not analyzed. In addition, in consideration of the immaturity of the coagulation system in children, especially newborns, more research is needed on SIC in children with sepsis. Secondly, our models screen out patients with a high risk of SIC but do not indicate who will benefit from anticoagulant therapy. It is still up to clinicians to decide whether to administer anticoagulant agents. However, the process from sepsis to severe coagulopathy is a continuous condition arising from a coagulation disorder. Early and accurate prediction of SIC can provide more time for clinicians to adjust treatment strategies, and study the potential effect of anticoagulant therapy in the early stage. Thirdly, this is a retrospective observational study. Missing data and input errors exist, despite the very high quality of the MIMIC-IV and eICU-CRD databases. Therefore, prospective validation is still required in the future. Compared with septic shock, for which advances have been made in recent years, giving rise to significant survival improvements, there is still a long way to go in the diagnosis and management of sepsis-associated coagulopathy.

## Conclusions

In conclusion, the present study developed two variants of the CatBoost model, which can discriminate septic patients who would and would not develop SIC.

## Data Availability Statement

Publicly available datasets were analyzed in this study. This data can be found here: https://mimic-iv.mit.edu/; https://eicu-crd.mit.edu/.

## Ethics Statement

The study was an analysis of two third-party anonymized publicly available databases with pre-existing institutional review board (IRB) approval.

## Author Contributions

Q-YZ, L-PL, and J-CL: conception and design. RG, G-WT, and ZL: administrative support. Q-YZ: collection and assembly of data. Q-YZ and L-PL: data analysis and interpretation. All authors: manuscript writing and final approval of manuscript.

## Conflict of Interest

The authors declare that the research was conducted in the absence of any commercial or financial relationships that could be construed as a potential conflict of interest.

## Supplementary Material

Supplementary Figure 1 Model performance in different patient cohorts in MIMIC-IV.

Supplementary Figure 2 Model interpretation of the full model in eICU-CRD.

Supplementary Figure 3 Model interpretation of the compact model in eICU-CRD.

Supplementary Table 1 Sepsis-induced coagulopathy (SIC) criteria.

Supplementary Table 2 Predictors extracted in MIMIC-IV and eICU-CRD.

Supplementary Table 3 Baseline characteristics between the SIC and non-SIC groups in the MIMIC-IV cohort.

Supplementary Table 4 Hyperparameter search domain in Bayesian optimization and final settings.

Supplementary Table 5 Results of logistic regression.
