# Supplementary material for: A Machine-Learning Approach for Dynamic Prediction of Sepsis-Induced Coagulopathy in Critically Ill Patients With Sepsis
Source: Front Med (Lausanne). 2021 Jan 21;7:637434. doi: 10.3389/fmed.2020.637434 (PMC7859637; doi:10.3389/fmed.2020.637434)
Supplement: Supplementary Figure 1 — Model performance in different patient cohorts in MIMIC-IV. [file Data_Sheet_1.docx]

Table S1. Sepsis‐induced coagulopathy (SIC) criteria

|  | Score | Range |
| --- | --- | --- |
| Platelet count (−10^9^/L) | 2 | < 100 |
|  | 1 | ≧ 100, < 150 |
| INR | 2 | > 1.4 |
|  | 1 | > 1.2, ≦1.4 |
| SOFA score | 2 | ≧ 2 |
|  | 1 | 1 |
| Total score for SIC |  | ≧ 4 |

SOFA score is the sum of 4 items (respiratory SOFA, cardiovascular SOFA, hepatic SOFA, renal SOFA).

Abbreviations: SIC sepsis‐induced coagulopathy, INR international normalized ratio, SOFA sequential organ failure assessment.

Table S3. Baseline characteristics between the SIC and non-SIC groups in the MIMIC-IV cohort

| Variables | | SIC (n=6744) | non-SIC (n=4618) | P-Value |
| --- | --- | --- | --- | --- |
| Demographic variables | |  |  |  |
|  | Age, median [Q1,Q3] | 67.87 [57.02,78.66] | 69.05 [56.92,80.76] | <0.001 |
|  | Male, n (%) | 3770 (55.90) | 2148 (46.51) | <0.001 |
|  | BMI, mean (SD) | 29.50 (8.35) | 29.39 (9.82) | 0.592 |
| Admission type, n (%) | |  |  | <0.001 |
|  | Elective | 96 (1.42) | 62 (1.34) |  |
|  | Emergency | 4186 (62.07) | 3070 (66.48) |  |
|  | Urgent | 1560 (23.13) | 812 (17.58) |  |
|  | Other | 902 (13.37) | 674 (14.60) |  |
| Ethnicity, n (%) | |  |  | 0.243 |
|  | Asian | 167 (2.48) | 143 (3.10) |  |
|  | Black | 739 (10.96) | 525 (11.37) |  |
|  | Hispanic | 271 (4.02) | 175 (3.79) |  |
|  | White | 4551 (67.48) | 3062 (66.31) |  |
|  | Other/Unknown | 1016 (15.07) | 713 (15.44) |  |
| Comorbidities, n (%) | |  |  |  |
|  | Hypertension | 3837 (56.90) | 2724 (58.99) | 0.028 |
|  | DM | 1405 (20.83) | 820 (17.76) | <0.001 |
|  | COPD | 818 (12.13) | 576 (12.47) | 0.604 |
|  | CHF | 2199 (32.61) | 1148 (24.86) | <0.001 |
|  | MI | 748 (11.09) | 341 (7.38) | <0.001 |
|  | CKD | 1948 (28.88) | 961 (20.81) | <0.001 |
|  | Leukemia | 172 (2.55) | 35 (0.76) | <0.001 |
|  | Strokes | 478 (7.09) | 627 (13.58) | <0.001 |
|  | Cancer | 1432 (21.23) | 747 (16.18) | <0.001 |
|  | Liver disease | 2033 (30.15) | 296 (6.41) | <0.001 |
| Severity of illness, median [Q1,Q3] | |  |  |  |
|  | SAPS-II (ICU admission) | 44 [35,54] | 37 [30,45] | <0.001 |
|  | SOFA (1st 24h) | 6 [4,9] | 4 [3,5] | <0.001 |
|  | GCS (1st 24h) | 15 [14,15] | 14 [13,15] | <0.001 |
| Vital signs | |  |  |  |
|  | Heart rate (/min), mean (SD) | 92.04 (18.15) | 87.92 (16.23) | <0.001 |
|  | Respiratory rate (/min), mean (SD) | 20.96 (4.65) | 20.41 (4.38) | <0.001 |
|  | MAP (mmHg), mean (SD) | 74.19 (10.59) | 78.29 (12.20) | <0.001 |
|  | Temperature (°C), mean (SD) | 36.93 (0.75) | 37.07 (0.64) | <0.001 |
| Laboratory tests | |  |  |  |
|  | INR, median [Q1,Q3] | 1.55 [1.30,2.00] | 1.20 [1.10,1.30] | <0.001 |
|  | PT (s), median [Q1,Q3] | 16.85 [14.30,21.81] | 13.00 [11.90,14.10] | <0.001 |
|  | Platelet (×10^9^/L), median [Q1,Q3] | 149.00 [92.33,234.10] | 243.00 [187.50,324.00] | <0.001 |
|  | RDW (%), mean (SD) | 16.71 (2.76) | 15.32 (2.14) | <0.001 |
|  | WBC (×10^9^/L), median [Q1,Q3] | 11.85 [7.70,17.60] | 11.95 [8.65,16.35] | 0.091 |
|  | pH, mean (SD) | 7.36 (0.09) | 7.39 (0.08) | <0.001 |
|  | PaO_2_ (mmHg), median [Q1,Q3] | 113.00 [87.00,159.43] | 109.00 [83.90,155.00] | 0.005 |
|  | PaCO_2_ (mmHg), median [Q1,Q3] | 39.00 [34.00,45.25] | 41.00 [35.63,48.75] | <0.001 |
|  | FiO_2_ (%), median [Q1,Q3] | 54.37 [45.00,75.00] | 50.00 [40.00,67.92] | <0.001 |
|  | PaO_2_/FiO_2_, median [Q1,Q3] | 226.21 [156.21,308.00] | 234.00 [166.54,313.75] | 0.007 |
|  | BE (mEq/L), mean (SD) | -2.12 (5.88) | 0.68 (5.41) | <0.001 |
|  | Bicarbonate (mmol/L), mean (SD) | 22.38 (5.35) | 24.54 (5.15) | <0.001 |
|  | Total CO_2_ (mEq/L), mean (SD) | 23.82 (6.42) | 26.92 (6.58) | <0.001 |
|  | Hematocrit (%), mean (SD) | 30.23 (5.85) | 31.73 (5.76) | <0.001 |
|  | Hemoglobin (g/dl), mean (SD) | 9.82 (1.94) | 10.33 (1.98) | <0.001 |
|  | Chloride (mmol/L), mean (SD) | 103.22 (7.28) | 103.41 (6.92) | 0.164 |
|  | Calcium (mmol/L), mean (SD) | 8.11 (1.26) | 8.29 (1.13) | <0.001 |
|  | Potassium (mmol/L), mean (SD) | 4.20 (0.67) | 4.17 (0.66) | 0.008 |
|  | Lactate (mmol/L), median [Q1,Q3] | 2.00 [1.35,3.27] | 1.30 [1.00,1.90] | <0.001 |
|  | Glucose (mmol/L), mean (SD) | 147.31 (67.94) | 150.29 (73.69) | 0.030 |
| Fluid balance | |  |  |  |
|  | Urine output (ml), median [Q1,Q3] | 790 [300,1545] | 1205 [605,2015] | <0.001 |
| Drug (1st 24h), n (%) | |  |  |  |
|  | Heparin | 2195 (32.55) | 1999 (43.29) | <0.001 |
|  | Linezolid | 192 (2.85) | 79 (1.71) | <0.001 |
|  | Vancomycin | 3751 (55.62) | 2122 (45.95) | <0.001 |
| Num of antibiotic types (1st 24h), n (%) | |  |  | <0.001 |
|  | **0** | 1188 (17.62) | 1111 (24.06) |  |
|  | **1** | 1459 (21.63) | 1219 (26.40) |  |
|  | **2** | 1814 (26.90) | 1179 (25.53) |  |
|  | **3** | 1338 (19.84) | 718 (15.55) |  |
|  | **>=4** | 945 (14.01) | 391 (8.47) |  |
| Support (1st 24h), n (%) | |  |  |  |
|  | CRRT (1st 24h) | 334 (4.95) | 28 (0.61) | <0.001 |
|  | Vaso (1st 24h) | 3158 (46.83) | 1070 (23.17) | <0.001 |
|  | MV (1st 24h) | 3390 (50.27) | 1874 (40.58) | <0.001 |
| Outcome | |  |  |  |
|  | Hosp. LOS (day), median [Q1,Q3] | 14.44 [7.88,26.74] | 10.89 [6.49,19.52] | <0.001 |
|  | ICU LOS (day), median [Q1,Q3] | 6.34 [2.94,13.03] | 4.00 [2.10,8.94] | <0.001 |
|  | 28-day mortality, n (%) | 1823 (27.03) | 497 (10.76) | <0.001 |

Abbreviations: SIC sepsis-induced coagulopathy, BMI body mass index, SD standard deviation, DM diabetes mellitus, COPD chronic obstructive pulmonary disease, CHF congestive heart failure, MI myocardial infarction, CKD chronic kidney disease, ICU intensive care unit, SAPS-II simplified acute physiology score II, SOFA sequential organ failure assessment, GCS Glasgow Coma Scale, MAP mean arterial pressure, BUN blood urea nitrogen, INR international normalized ratio, PT prothrombin time, RDW red cell distribution width, WBC white blood cell count, pH potential hydrogen, PaO_2_ partial pressure of oxygen, PaCO_2_ partial pressure of carbon dioxide in arterial blood, FiO_2_ fraction of inspiration O_2_, BE base excess, CRRT continuous renal replacement therapy, Vaso vasopressor, MV mechanical ventilation, Hosp. hospital, LOS length of stay.

Table S4. Hyperparameter search domain in Bayesian Optimization and final settings

| Hyperparameters | Search domain | Final setting |
| --- | --- | --- |
| 'depth' | [2, 8] (integer) | 3 |
| 'bagging_temperature' | [1, 50] | 44.2 |
| 'reg_lambda' | [1, 200] | 106.6 |
| 'learning_rate' | [0.01, 0.2] | 0.081 |
| 'min_data_in_leaf' | [1, 3] | 1 |

Table S5. Results of Logistic Regression

|  | **Coef.** | **OR** | **95% CI** | **Std.Err.** | **z** | **P>\|z\|** |
| --- | --- | --- | --- | --- | --- | --- |
| **Hypertension** | 0.289 | 0.749 | [0.699, 0.803] | 0.035 | 8.236 | <0.001 |
| **DM** | -0.048 | 1.049 | [0.969, 1.135] | 0.040 | -1.183 | 0.237 |
| **COPD** | 0.270 | 0.763 | [0.708, 0.824] | 0.039 | 6.943 | <0.001 |
| **CHF** | 0.188 | 0.829 | [0.770, 0.893] | 0.038 | 4.938 | <0.001 |
| **MI** | -0.037 | 1.038 | [0.936, 1.151] | 0.053 | -0.711 | 0.477 |
| **CKD** | -0.112 | 1.119 | [1.036, 1.209] | 0.040 | -2.840 | 0.005 |
| **Leukemia** | -1.339 | 3.815 | [2.989, 4.865] | 0.124 | -10.779 | <0.001 |
| **Strokes** | -0.705 | 2.024 | [1.815, 2.255] | 0.055 | -12.724 | <0.001 |
| **Cancer** | 0.437 | 0.646 | [0.600, 0.696] | 0.038 | 11.526 | <0.001 |
| **Liver disease** | -0.224 | 1.251 | [1.133, 1.383] | 0.051 | -4.420 | <0.001 |
| **Age** | 0.012 | 0.988 | [0.986, 0.991] | 0.001 | 9.176 | <0.001 |
| **Gender** | 0.083 | 0.920 | [0.860, 0.984] | 0.034 | 2.434 | 0.015 |
| **BMI** | 0.004 | 0.996 | [0.993, 0.999] | 0.002 | 2.662 | 0.008 |
| **Admission type 1** | -0.246 | 1.279 | [1.185, 1.380] | 0.039 | -6.333 | <0.001 |
| **Admission type 2** | 0.472 | 0.624 | [0.523, 0.744] | 0.090 | 5.252 | <0.001 |
| **Admission type 3** | -0.241 | 1.273 | [1.175, 1.379] | 0.041 | -5.912 | <0.001 |
| **Ethnicity 1** | 0.202 | 0.817 | [0.738, 0.904] | 0.052 | 3.903 | <0.001 |
| **Ethnicity 2** | -0.974 | 2.649 | [2.010, 3.490] | 0.141 | -6.927 | <0.001 |
| **Ethnicity 3** | 0.707 | 0.493 | [0.435, 0.559] | 0.064 | 10.980 | <0.001 |
| **Ethnicity 4** | -0.652 | 1.919 | [0.842, 4.371] | 0.420 | -1.551 | 0.121 |
| **Ethnicity 5** | -0.017 | 1.017 | [0.933, 1.108] | 0.044 | -0.388 | 0.698 |
| **SAPS-II** | 0.010 | 0.990 | [0.988, 0.993] | 0.001 | 7.646 | <0.001 |
| **GCS** | 0.035 | 0.966 | [0.947, 0.985] | 0.010 | 3.529 | <0.001 |
| **Heart rate** | 0.007 | 0.993 | [0.991, 0.995] | 0.001 | 6.189 | <0.001 |
| **Respiratory rate** | 0.004 | 0.996 | [0.974, 1.018] | 0.011 | 0.339 | 0.735 |
| **MAP** | -0.005 | 1.005 | [1.003, 1.008] | 0.001 | -3.774 | <0.001 |
| **SpO2** | 0.026 | 0.974 | [0.959, 0.990] | 0.008 | 3.210 | 0.001 |
| **Temperature** | -0.073 | 1.076 | [1.021, 1.133] | 0.026 | -2.748 | 0.006 |
| **Plateau pressure** | 0.013 | 0.987 | [0.979, 0.994] | 0.004 | 3.464 | 0.001 |
| **Tidal volume** | 0.000 | 1.000 | [0.999, 1.000] | 0.000 | 1.003 | 0.316 |
| **PEEP** | -0.009 | 1.009 | [0.996, 1.022] | 0.006 | -1.376 | 0.169 |
| **RSBI** | -0.002 | 1.002 | [0.997, 1.007] | 0.003 | -0.914 | 0.361 |
| **Albumin** | 0.185 | 0.831 | [0.732, 0.944] | 0.065 | 2.853 | 0.004 |
| **Total protein** | -0.219 | 1.245 | [1.100, 1.408] | 0.063 | -3.476 | 0.001 |
| **Anion gap** | 0.011 | 0.989 | [0.975, 1.003] | 0.007 | 1.514 | 0.130 |
| **BUN** | -0.007 | 1.007 | [1.005, 1.008] | 0.001 | -8.538 | <0.001 |
| **Creatinine** | 0.150 | 0.861 | [0.836, 0.886] | 0.015 | 10.138 | <0.001 |
| **Globulin** | 0.094 | 0.910 | [0.803, 1.033] | 0.064 | 1.467 | 0.142 |
| **Fibrinogen** | 0.000 | 1.000 | [1.000, 1.000] | 0.000 | -0.105 | 0.916 |
| **INR** | 0.179 | 0.836 | [0.616, 1.135] | 0.156 | 1.147 | 0.251 |
| **PT** | -0.009 | 1.009 | [0.980, 1.039] | 0.015 | -0.597 | 0.551 |
| **PTT** | 0.008 | 0.992 | [0.990, 0.993] | 0.001 | 10.545 | <0.001 |
| **ALT** | -0.001 | 1.001 | [1.000, 1.002] | 0.000 | -3.568 | <0.001 |
| **AST** | 0.001 | 0.999 | [0.999, 1.000] | 0.000 | 3.228 | 0.001 |
| **ALP** | 0.000 | 1.000 | [1.000, 1.001] | 0.000 | -4.369 | <0.001 |
| **Total bilirubin** | 0.106 | 0.899 | [0.829, 0.976] | 0.042 | 2.536 | 0.011 |
| **Direct bilirubin** | -0.101 | 1.106 | [1.005, 1.219] | 0.049 | -2.052 | 0.040 |
| **Indirect bilirubin** | -0.057 | 1.059 | [0.935, 1.198] | 0.063 | -0.897 | 0.370 |
| **MCH** | -0.305 | 1.357 | [1.146, 1.606] | 0.086 | -3.541 | <0.001 |
| **MCHC** | 0.261 | 0.770 | [0.660, 0.899] | 0.079 | 3.293 | 0.001 |
| **MCV** | 0.092 | 0.912 | [0.865, 0.961] | 0.027 | 3.442 | 0.001 |
| **Platelet** | -0.001 | 1.001 | [1.000, 1.001] | 0.000 | -5.815 | <0.001 |
| **RBC** | 0.089 | 0.915 | [0.591, 1.418] | 0.223 | 0.397 | 0.691 |
| **RDW** | 0.034 | 0.967 | [0.953, 0.981] | 0.007 | 4.614 | <0.001 |
| **WBC** | 0.000 | 1.000 | [0.996, 1.004] | 0.002 | 0.181 | 0.857 |
| **pH** | -1.091 | 2.977 | [0.541, 16.395] | 0.871 | -1.253 | 0.210 |
| **PaO2** | -0.001 | 1.001 | [0.999, 1.002] | 0.001 | -0.996 | 0.319 |
| **PaCO2** | -0.014 | 1.014 | [1.002, 1.025] | 0.006 | -2.356 | 0.018 |
| **FiO2** | -0.003 | 1.003 | [0.994, 1.011] | 0.004 | -0.631 | 0.528 |
| **PaO2/FiO2** | 0.001 | 0.999 | [0.999, 1.000] | 0.000 | 1.648 | 0.099 |
| **SO2** | -0.002 | 1.002 | [0.996, 1.008] | 0.003 | -0.594 | 0.553 |
| **BE** | -0.005 | 1.005 | [0.957, 1.055] | 0.025 | -0.200 | 0.842 |
| **Bicarbonate** | -0.030 | 1.030 | [1.018, 1.043] | 0.006 | -4.870 | <0.001 |
| **AADO2** | 0.001 | 0.999 | [0.998, 1.001] | 0.001 | 0.998 | 0.318 |
| **Total CO2** | 0.041 | 0.960 | [0.920, 1.001] | 0.021 | 1.913 | 0.056 |
| **Hematocrit** | -0.002 | 1.002 | [0.950, 1.058] | 0.027 | -0.087 | 0.930 |
| **Hemoglobin** | 0.003 | 0.997 | [0.836, 1.188] | 0.090 | 0.037 | 0.971 |
| **Chloride** | 0.001 | 0.999 | [0.988, 1.010] | 0.006 | 0.195 | 0.845 |
| **Calcium** | -0.011 | 1.011 | [0.983, 1.039] | 0.014 | -0.765 | 0.444 |
| **Potassium** | 0.006 | 0.994 | [0.942, 1.048] | 0.027 | 0.231 | 0.817 |
| **Sodium** | 0.009 | 0.991 | [0.980, 1.002] | 0.006 | 1.529 | 0.126 |
| **Lactate** | 0.062 | 0.940 | [0.908, 0.971] | 0.017 | 3.666 | <0.001 |
| **Glucose** | 0.000 | 1.000 | [1.000, 1.001] | 0.000 | -2.438 | 0.015 |
| **SpO2/FiO2** | 0.065 | 0.937 | [0.728, 1.208] | 0.129 | 0.500 | 0.617 |
| **ROX index** | -2.705 | 14.954 | [0.668, 334.956] | 1.586 | -1.705 | 0.088 |
| **Urine output** | 0.000 | 1.000 | [1.000, 1.000] | 0.000 | -0.259 | 0.796 |
| **SIC platelet** | 0.539 | 0.583 | [0.541, 0.629] | 0.039 | 13.989 | <0.001 |
| **SIC INR** | 0.332 | 0.717 | [0.682, 0.755] | 0.026 | 12.796 | <0.001 |
| **Respiratory SOFA** | 0.014 | 0.986 | [0.955, 1.017] | 0.016 | 0.893 | 0.372 |
| **Liver SOFA** | 0.074 | 0.929 | [0.868, 0.994] | 0.035 | 2.141 | 0.032 |
| **Renal SOFA** | 0.046 | 0.955 | [0.928, 0.984] | 0.015 | 3.064 | 0.002 |
| **Cardiovascular SOFA** | -0.032 | 1.033 | [0.999, 1.068] | 0.017 | -1.887 | 0.059 |
| **SIC days** | 0.083 | 0.920 | [0.915, 0.926] | 0.003 | 28.877 | <0.001 |
| **RBC Transfusion** | 0.000 | 1.000 | [1.000, 1.000] | 0.000 | -1.207 | 0.228 |
| **FFP Transfusion** | 0.000 | 1.000 | [0.999, 1.000] | 0.000 | 0.977 | 0.329 |
| **PLT Transfusion** | 0.002 | 0.998 | [0.997, 0.999] | 0.001 | 3.496 | <0.001 |
| **Heparin use** | -0.133 | 1.142 | [1.068, 1.221] | 0.034 | -3.890 | <0.001 |
| **Heparin days** | -0.013 | 1.013 | [1.009, 1.017] | 0.002 | -6.694 | <0.001 |
| **Antibiotic types** | 0.074 | 0.929 | [0.900, 0.957] | 0.015 | 4.823 | <0.001 |
| **Linezolid** | 0.039 | 0.962 | [0.821, 1.127] | 0.081 | 0.478 | 0.633 |
| **Vancomycin** | -0.209 | 1.232 | [1.141, 1.330] | 0.039 | -5.359 | <0.001 |
| **CRRT use** | 0.085 | 0.919 | [0.817, 1.031] | 0.060 | 1.432 | 0.152 |
| **Vasopressor use** | 0.179 | 0.836 | [0.764, 0.915] | 0.046 | 3.891 | <0.001 |
| **MV durations** | -0.001 | 1.001 | [1.001, 1.001] | 0.000 | -12.630 | <0.001 |

Admission type 0: Emergency (reference); Admission type 1: Urgent; Admission type 2: Elective; Admission type 3: Other; Ethnicity 0: White (reference); Ethnicity 1: Black; Ethnicity 2: Asian; Ethnicity 3: Hispanic; Ethnicity 4: Native; Ethnicity 5: Other/Unknown

Fig S1. Model performance in different patient cohorts in MIMIC-IV

Different validation sets were derived based on SPAS-II (A), age (B), ethnicity (C) and time since sepsis onset (D). AUC of the full and the compact models in each set was measured by using bootstrap method. The colored area represents 95% confidence intervals.

Abbreviations: Full the full model, Comp the compact model, AUC area under receiver operating characteristic curve, SAPS-II Simplified Acute Physiology Score-IV.

Fig S2. Model interpretation of the full model in eICU-CRD

Fig S3. Model interpretation of the compact model in eICU-CRD
